# Healthcare leaders and professionals’ perspectives of the ICON programme to prevent abusive head trauma in infants: a qualitative study

**DOI:** 10.1186/s12889-025-24682-0

**Published:** 2025-10-21

**Authors:** Julie M. Brose, Julie Mytton, Mark D. Lyttle, Maria Theresa Redaniel, Jelena Savović, Hugh McLeod, Carlos Sillero Rejon, Joni Jackson, Maria Barnes

**Affiliations:** 1 Population Health Sciences, Bristol Medical School, University of Bristol, Bristol, UK; 2 The National Institute of Health and Care Research Applied Research Collaboration West (NIHR ARC West) at University Hospitals Bristol and Weston NHS Foundation Trust, Bristol, UK; 3 Perron Institute of Neurological and Translational Sciences, University of Western Australia, Perth, Australia; 4 School of Health and Social Wellbeing, University of the West of England, Bristol, UK; 5 Emergency Department, Bristol Royal Hospital for Children, Bristol, UK; 6 Research in Emergency Care Avon Collaborative Hub (REACH), University of the West of England, Bristol, UK; 7 National Cancer Registry Ireland, Cork, Ireland

**Keywords:** Abusive head trauma, Prevention programme, Shaken baby syndrome, Programme evaluation, Infants

## Abstract

**Background:**

Abusive head trauma (AHT) in infants is the most common abusive injury in young children, and increased awareness has resulted in the development of prevention programmes. Most research evaluating AHT prevention programmes report parental and carer perspectives. Little is known about barriers and facilitators to adopting, implementing, and maintaining educational programmes from the perspectives of managers and staff delivering the education. ICON is an AHT prevention programme currently being delivered in National Health Service hospital and primary care settings in the United Kingdom.

**Methods:**

This study evaluated the ICON programme from the perspective of managers and healthcare professionals through the RE-AIM framework using qualitative methods. Fifty-three managers and healthcare professionals across six geographical areas in England participated in individual interviews and focus groups between October 2022 and April 2023. Data collection and analysis were concurrent, systematic, and iterative, using framework analysis as a guide to explore factors impacting ICON’s reach and the key enablers and obstacles to its effectiveness, adoption, implementation, and maintenance.

**Results:**

Four primary enablers and related challenges to the ICON programme’s impact were identified. Fidelity to the programme’s recommended touchpoints and message impacted ICON’s reach to new parents and carers. Parental receptiveness to the programme was affected by staff individualising their approach. Staff buy-in was related to staff workload and previous experiences with AHT. Managers with strategic leadership responsibility for reducing infant mortality and able to provide governance oversight fostered successful adoption, implementation, and maintenance of the programme.

**Conclusions:**

Staff are willing and able to deliver the ICON programme, including, where necessary, delivering the key messages in a format acceptable to families varying situations, if given the workload and training to do so. Those in leadership positions influence the likelihood of successful adoption, delivery and longer-term mainstreaming, if they are able to prioritise the programme. Understanding the barriers and facilitators to ICON’s delivery has the potential to inform policy by facilitating the uptake of the programme by settings, enabling delivery of ICON to reach the needs of local families, and ensuring sustainability of the ICON programme.

**Supplementary Information:**

The online version contains supplementary material available at 10.1186/s12889-025-24682-0.

## Background

Abusive head trauma (AHT) is the most common abusive injury in infants, with an incidence of 20–24/100,000 in the United Kingdom [1]. The consequences of violent shaking (commonly referred to as shaken baby syndrome) or blunt trauma include neurological impairment or death [2]. The age at which AHT most commonly occurs is chronologically matched to the peak in infant crying, leading to the hypothesis that there may be a causal association [3]. Over the past 30 years, increased awareness has resulted in the development of prevention programmes, which aim to reduce AHT by changing behaviour through education for carers (e.g., on coping strategies), healthcare professionals (e.g., on diagnosis or prevention), or broader community public health campaigns (e.g., educating high schoolers or mass media campaigns for the general public). Examples include the Period of PURPLE Crying, Perinatal Shaken Baby Syndrome Prevention Program (PSBSPP), or the ICON programme [4–8]. However, studies have reported mixed findings for their impact, with some questioning their effectiveness [4, 9–11] and others describing the positive impact of the programme [5, 6, 12, 13].

Evaluating the effectiveness of such public health campaigns is challenging due to variations in their content, implementation and management, as well as the multifactorial nature of behaviour change. Effectiveness is also influenced by programme characteristics, individual staff, the healthcare system, and carer-related factors. Most studies evaluating these programmes focus on the reach and effectiveness on the parent or carer [6, 12, 13], while few explore the perspectives of healthcare professionals who either deliver the programme or are in decision-making roles influencing how the programme is managed locally.

The ICON programme was developed in the United Kingdom to reduce AHT incidence through educating parents and carers [7]. ICON has four key messages: (i) Infant crying is normal, (ii) Comforting strategies work, (iii) it is Okay to walk away from your baby, and (iv) Never ever shake a baby [14]. The programme aims to normalise crying as part of infant development, and to reassure and provide coping strategies for carers. Five main touchpoints are recommended postnatally: in hospital before discharge, within the first ten days at home, two weeks, three weeks, and at the routine six-to-eight week baby health check. ICON specifies that the message should be delivered by the midwife, health visitor, and primary care physician (GP). The ICON programme has multiple physical and online resources including leaflets printed in several languages, stickers and fridge magnets, a GP checklist, videos, coping with crying plans, infographics, and patient-facing posters [7].

Of the published studies reporting HCP views on AHT prevention programmes, one noted that nurses liked the training and materials but did not explore factors related to implementation or management [5]. Another documented nurses’ perceptions of facilitators and barriers to implementing a locally developed AHT intervention, commenting on factors pertaining to individual nurses, new parents, the hospital system, and the guidelines [15]. The authors found that barriers to the programme included high workload (staff), language barriers (parents), and limited support or resources (hospitals/guidelines). Facilitators included embedding the education in routine activities (staff), receptiveness (parents), sufficient resources and leadership (hospitals), and ensuring the availability of brochures and mandatory education to parents (guidelines) [15]. Less is known about how well programmes reach all carers eligible to receive the programme, the effectiveness of the programme to reduce AHT or to help carers cope with persistent crying, or how those factors are affected by the way the programme is taken up by local services and managed. As part of a larger mixed methods study, we explored the barriers and facilitators to these factors during a qualitative evaluation of the ICON programme from the perspectives of staff in leadership positions and HCPs.

## Methods

### Study aim

We used qualitative methods and the RE-AIM Framework to identify key barriers and facilitators to the ICON programme in practice [16, 17]. The RE-AIM Framework’s emphasis on programme evaluation assisted with identifying barriers and facilitators to behavioural interventions, such as ICON’s message of coping strategies to assist new parents in caring for a crying infant. The research question for this qualitative component of the larger mixed-methods study was as follows: Amongst managers and HCPs, what factors determine the reach of the ICON programme, the ability to be effective, and the key enablers and obstacles to its adoption, implementation, and maintenance?

### Lived experience panel

A lived-experience panel comprising seven parents of young children from diverse backgrounds across the greater Bristol area was recruited to help guide the research design and analysis. Prior to study commencement, two online meetings and several email communications were held with the lived experience panel and team members (MB, ML). Feedback informed recruitment routes and materials, patient information and consent forms, and topic guides. In line with panel recommendations, the recruitment advert and participant information sheet were clarified and shortened. Following data analysis, a stakeholder event with managers and HCPs was held to discuss findings, providing an opportunity to sense-check and validate themes.

### Participants and recruitment

Six geographic areas across England were purposively chosen to represent northern, central, and southern England, in rural and urban areas, with differing regional deprivation and ethnic diversity. ICON delivery leads from these six areas were invited to participate, and study information was cascaded to team leads and individual HCPs. Professionals were eligible to participate if they were responsible for commissioning, implementing, or delivering ICON messages. Professionals included commissioners, safeguarding leads, managers of health visiting and midwifery teams (collectively ‘managers’), health visitors, midwives, and GPs (collectively ‘HCPs’).

Online expression of interest (EOI) forms were completed using Research Electronic Data Capture (REDCap) software hosted on University of Bristol servers, or by contacting the researcher (JB) directly [18]. Purposive sampling of EOIs ensured a range of roles and experiences were reflected across geographic areas. Staff who expressed interest were sent a participant information sheet and signposted to a study website containing further detail. Prior to each interview or focus group, JB discussed study information and provided space for questions, following which participants provided written consent. Participants also completed an electronic demographic survey. No incentives for participation were offered. Study sample size was determined based on data saturation.

### Data collection and analysis

#### Interview guide

The topic guide was developed to address the study aims, informed by existing information on the ICON programme, and the RE-AIM Framework [7, 19]. Questions were designed to elicit discussions on factors influencing ICON’s reach and effectiveness, and barriers and facilitators to its adoption, implementation and maintenance in practice. Validity of the topic guide was strengthened by trialling it and by seeking feedback from a lived-experience panel. Due to their unique roles, a separate interview guide was created for managers and HCPs delivering ICON (Additional file 1).

#### Data collection

Data collection occurred through individual interviews and focus groups conducted by JB between October 2022 to April 2023. Staff in leadership roles were invited to participate in individual interviews, though some leadership teams requested a focus group. HCP teams primarily requested focus groups due to work scheduling but could request an individual interview if preferred. Interviews and focus groups were conducted online through Zoom or Microsoft Teams, with content audio-recorded on a password-protected device; no video recording occurred. A confidential transcription service was used, and all transcripts were pseudonymised.

#### Data analysis

Data collection and analysis were concurrent, systematic, and iterative. Initial interviews were transcribed and read in-depth to identify and isolate items of interest and conduct the initial coding using thematic framework analysis (JB). Framework analysis has increasingly been used in interdisciplinary applied health research since its inception in social policy research [20], and enabled comparison of data across participant groups in our study. An analytical framework to code further transcripts was created using the RE-AIM framework. NVivo 12 was used to manage data. Two experienced qualitative researchers (JB, MB) then conducted a coding comparison exercise to refine codes, discussing any discrepancies in coding and ensuring consensus was reached. As data analysis progressed, further discussions occurred to enhance the reliability and validity of data analysis by involving an expert in the RE-AIM framework (JM), and a clinician experienced in managing AHT (ML).

## Findings

### Participants

Fifty-three professionals participated; 19 were in leadership positions on integrated care boards, managers, commissioners, or designated nurse or GP safeguarding leads, whilst 34 were HCPs including health visitors, midwives (community, hospital, and substance abuse specialists), nurses (in the neonatal intensive care unit [NICU], community oxygen programme, and specialists for young mums), feeding specialists (dietician and breastfeeding support worker), or GPs. Of the 19 in leadership positions, all had one to five years of involvement in overseeing the ICON message in hospital, community, or primary care settings. The 34 HCPs had between eight months and 30 years of clinical experience.

Although the initial study protocol anticipated 30 participants in leadership roles or HCPs delivering ICON, additional participants were sought to ensure representation from multiple disciplines and geographic areas. The 53 participants ensured a diverse range of perspectives, practices and geographical regions were captured across multiple professional groups (Table 1). One researcher (JB) conducted 12 individual interviews and three focus groups for managers, and six individual interviews and eight focus groups for HCPs.

**Table 1:** Participant characteristics

Participant characteristics	Number of respondents
Profession^1^	
Health visitor	21
Manager	11
Nurse	9
Midwife	5
Physician	4
Allied health (e.g., dietician, social worker)	3
Setting^2, 3^	
Community	32
Hospital	11
Primary care	8
Other	10
Experience^3^	
0-23 months	5
2-4 years	13
5-9 years	15
>10 years	13
Age^3^	
25-34 years	4
35-44 years	11
45-54 years	14
55-64 years	17
>65 years	1
Gender	
Male	1
Female	52

(1) Staff in leadership positions could self-identify as manager or their professional role. (2) Staff could choose more than one option so the numbers may add to more than 100%. (3) Not all staff answered the question so numbers may not add up to 100%

### Results by RE-AIM dimension

Multiple themes were developed using the RE-AIM framework for analysis. Themes are presented under each RE-AIM heading and describe facilitators and barriers reported by managers and HCPs. A summary of the findings is listed in Table 2.

**Table 2:** Findings from professional qualitative evaluation mapped to RE-AIM

	RE-AIM concept	Managers	Health care professionals
Facilitators	Reach	Documentation processes	Prior knowledge and professional judgment used to individualise approaches
		Dissemination to relevant staff at recommended touchpoints	
	Effectiveness	Success rolling out ICON programme as a public health campaign	Parental normalisation of crying
			Fosters putting baby down when needed
		ICON as a tool to address serious case reviews	Individualised approach
	Adoption	Impetus from serious case reviews	Similar to existing messages
		Commissioner endorsement, financial support	Practical advice - easy message
		Governance structure, strategic role	Impetus from serious case reviews
		Bespoke implementation strategy	Resources to support adoption or roll-out
		Learning from other sites	
	Implementation	Business plan for touchpoints, message delivery	Material fosters discussion
			Touchpoints adhered to
			Technology
		ICON adjustments to fit population needs	Individualised approach
			Reinforced by positive feedback
		Involvement of non-health services	
	Maintenance	Strategic ownership; steering group	ICON embedded in practice
			Managerial support
		Training plan	Material available, ICON week
Barriers	Reach	Reaching all target populations equally	Reaching all target populations
			Mums in sharing information role
			Disconnect in HCP expectations
	Effectiveness	Challenge assessing effectiveness due to ICON’s preventative message	Parental medical concerns
			Parental contextual factors
			Limited parental adoption
			Workforce pressures
	Adoption	Training roll-out issues	Heavy workload
		Organisational challenges	Low staff morale
			GP hesitancy
	Implementation	High staff workload	No annual review/training
		Funding challenges	Challenges with materials
		Touchpoints not adhered to	Lack of confidence
			Lack of interprofessional agreement &/or understanding
	Maintenance	Limited strategic vision	No annual training
		Challenges embedding	Heavy workload/low morale
		Limited resources, training, plan	
			Touchpoints not adhered to

### Reach of the ICON programme

Reach was evaluated as the degree to which the ICON programme was received by HCPs and intended recipients, the carers of new babies. Managers described how they rolled out the ICON programme to relevant staff, who then provided the messages to new parents. Most managers said ICON messages were disseminated at relevant touchpoints, describing processes to target new parents. However, most midwives and GPs interviewed commented that they had observed team members from their own and other professions not delivering the ICON message consistently. This pattern was also highlighted by health visitors who described more consistent delivery in their own teams. Staff delivering ICON described individualised approaches, based on knowledge of the parent and their circumstances. For example, differentiating ICON from cry-it-out approaches can assist with reaching parents who feel guilt with putting their crying infant down in a safe space for a couple minutes if feeling overwhelmed. However, both managers and HCPs described challenges reaching all target populations such as non-English speaking parents, and reliance on mothers to discuss messages with fathers or other carers.

#### Facilitators

Managers described dissemination to HCPs using online and in-person training sessions. Many staff reported successful dissemination of the ICON message to new parents, emphasising the importance of infant safety. To encourage dissemination to fathers, documentation procedures were developed to capture who received ICON messages, such as a sticker on the child’s file (the ‘Red Book’) and an indication of who was present. Some HCPs intentionally sought out fathers to discuss ICON messages rather than relying on mothers to pass these on. With increasing numbers of people working from home due to COVID-19, HCPs described having more fathers present at postnatal appointments compared to previously. Despite this, most fathers still did not attend appointments.

*HCP29: Dad’s generally there at the new birth visit*,* generally not at the antenatal or six to eight week*,* but the new birth. So at least one contact family members or dad will be there… If we’re doing a targeted one and the dad’s upstairs*,* you can say to [him]*,* “Wanna come downstairs.” … I always include [the dads] in my assessment around emotional well–being as well. Because… we focus so much on the mums*,* you know*,* we forget the dads don’t go through it like physically*,* but they are emotionally.*

#### Barriers

The most common barrier reported by managers and HCPs related to reaching all target populations. Limited reach was often due to a primary focus on mothers, fathers infrequently attending appointments, and limited awareness and use of resources in other languages. As fathers or other carers were not often present during the appointments, staff routinely took a pragmatic approach and relied on mothers to pass information on to partners.

*HCP25: You always say*,* ‘Look*,* can you just make sure that you share that with your husband*,*’… I just normally say ‘it’s more common that dads lose their patience quicker than mums and they’re more likely to shake the baby. So*,* make sure you show him*,*’ but then you just hope that they would.*

HCPs spoke of rarely targeting foster parents, grandparents, or other carers. Although staff discussed the importance of prioritising high-risk parents, others described the need to ensure all new parents are given ICON messages. Some HCPs spoke of feeling uncomfortable with discussing ICON messages with certain parents:

*HCP19: I think [talking about shaken baby syndrome] makes you a little uncomfortable sometimes*,* and I think probably the most difficult parents [to talk to] … is parents who are already on our safeguarding list*,* or we already know there are issues there.*

### Effectiveness of the ICON programme

Effectiveness was viewed in terms of the perceived impact on parental knowledge and provision of coping for crying, both of which were described by managers and HCPs. Managers also described the challenge of evaluating effectiveness as messaging is preventative, and they were unable to undertake statistical analysis of the impact of ICON in terms head injuries or deaths avoided. Some participants reported rarely receiving feedback from parents unless strategies were unsuccessful, often from parents whose babies cried a lot or had other health concerns.

#### Facilitators

HCPs described how ICON provided a framework to discuss coping strategies with new parents in a simple message; one described ICON as *“an empowering tool”* (HCP 30). Staff highlighted ICON’s credibility was enhanced when discussed by HCPs, increasing parental willingness to implement it. Managers and HCPs perceived ICON benefitted new parents as they believed it reduced incidence of AHT or death. They described how messages normalised crying, reassured parents they were not at fault, provided coping strategies, and gave permission to put the baby in a safe place and take a breather. HCPs described how ICON gave parents *“that feeling of I’m not alone in this*,* I have some suggestions that I can feel confident about that I can try”* (HCP31).

*M3: We’ve got no hard data*,* but we had this run*,* I think it was of nine cases of serious NAI*,* Non-Accidental Injury*,* abusive head trauma in infants and what we saw after we’d done the ICON promotion*,* the following year our numbers dropped. Now*,* our numbers are only low*,* so you can’t really say it’s statistically significant*,* so it was more a feeling.*

Individualising ICON delivery was perceived by HCPs as important to optimise effectiveness. Prior knowledge and professional judgment facilitated individualised approaches, such as combining with other messages, or easing ICON in when discussing coping with crying. Some described addressing unrealistic parental expectations that babies should not cry, or guilt associated with putting the baby down if they needed a break. NICU nurses described tailoring messages to be relevant on discharge, transitioning from being quiet babies in the NICU with constant nursing supervision to crying at home with no hospital support. One community midwife described her communication strategies:

*HCP16: It’s finding the right vocabulary for the certain type of woman that you’re seeing. … We’re a completely diverse population of*,* you know*,* low income*,* high income*,* different ethnicities*,* different religions*,* different backgrounds. No woman is the same. You’re seeing everyone from all different areas. And I feel like between those first baby*,* tenth babies*,* do you know what I mean*,* between those women as a student and even as a midwife*,* you have to completely adapt the way you talk about it.*

HCPs described approaches for “higher-risk” parents or those known to safeguarding teams. For example, midwives described how they helped parents feel the message was for everyone and they were not being targeted:

*HCP23: I think I’m quite careful… I very much make it clear that this is something we say to everybody. I tend to use the phrase*,* ‘We’re not picking on you because of your addiction. We’re not picking on you because of your age. This is something that we’re telling everybody*,*’ because they can be quite traumatised families anyway.*

#### Barriers

Managers described challenges evaluating effectiveness as the focus is prevention. Effectiveness was believed to be related to several factors, including the context in which parents heard the messages. These included whether parent or baby was sleeping well, pre-existing mental health challenges, and support (from spouse, family, or friends). For example, a parent struggling with breastfeeding may focus on feeding concerns and not hear other messages.

*HCP34: You’ve got two patients. You’ve got the baby themself and the parent and their expectations*,* their experience of parenting – whether they’re first-time mums or dads or not – which influences their health-seeking behaviours and how anxious or concerned they are about their baby and what is or isn’t normal … often this can get wrapped up in postnatal depression*,* postnatal mental health as well being a big issue sometimes. That can obviously influence how challenging it is for new mums or new parents to deal with this.*

Some HCPs and GPs described as a barrier the belief of some parents that crying was due to a medical condition and not normal development. One GP described providing medication for babies at parental request due to this, despite explaining to the researcher that only time would help.

*HCP5: I don’t find that they readily accept that babies just cry. There’s got to be a reason. Have they got reflux? Have they got colic? Is it because they’re constipated? Whatever*,* whatever. I don’t think it’s easy to say to a parent*,* ‘I think we’ve exhausted absolutely everything*,* I think baby’s just fine*,*’ because parents are like*,* ‘no*,* no*,* no*,* no*,* there’s got to be a reason for that.*

HCPs reported that if they did not individualise approach in addressing contextual factors, parental concerns may go unaddressed and reduce ICON’s impact. In addition, challenges experienced by staff (such as high workloads) impacted whether the HCP had sufficient time to address parental receptiveness about ICON, affecting effectiveness with those parents.

### Adoption of the ICON programme

Adoption of ICON by management and HCPs was explored to better understand which groups take it up, and why. Staff accounts clustered around themes of facilitators or barriers to ICON adoption. All HCPs described the difference it made to practice, including normalisation of crying as part of development. However, HCPs reported that staff who did not adopt ICON were concerned about time constraints, high workloads, or not seeing the message as relevant or significant enough.

#### Facilitators

Experience of infant death or injury and resultant serious case review was an impetus for adopting ICON as a preventative measure. Managers exploring ICON often discussed adoption with sites where the practice was embedded, and described the importance of governance structures to facilitate successful adoption and roll-out, including writing a business case to obtain funding.

*M4: I think that probably the key thing was putting together a good quality business case and also then looking at the correlation between what was relatively low cost with the cost of*,* for example*,* a child who has catastrophic injuries and needs lifelong care as a result of abusive head trauma*,* so it was almost we were coming at it from various different approaches*,* you know*,* a spend to save in terms of the preventative side.*

Financial support and endorsement from clinical commissioning groups (CCG) facilitated adoption and roll-out. Strategic planning was essential for effective inter-agency coordination and consistent messaging. Managers described significant value from involving multiple stakeholders, including CCG leadership, safeguarding partnerships, healthcare managers, local authorities, the national ICON team, HCPs, and people living with the effects of AHT; some also engaged police, social services, or early childhood educators.

*M2: It is about getting that engagement at every tier*,* isn’t it? So*,* making sure that you have the support of senior leadership and the safeguarding board is probably a good way to get that because it’s a statutory body so it will exist in every area*,* and it brings together those key agencies. Then*,* getting people with a passion at the right level to be involved in the steering group*,* then setting a strategy and action plan around that*,* really. There’s a lot of information and support from the national team that can help with defining what the message is and providing the train the trainer information.*

Delivery of training programmes was often described as a smooth process. Each site’s training strategy facilitated ICON’s roll-out, with many describing bespoke implementation strategies and how they learned to do this effectively from other sites. Some used a train-the-trainer programme, others had one trainer for all education sessions, and others conducted online training; having these varied training options facilitated roll-out.

HCPs were more likely to adopt ICON if they had previous experience of AHT. One HCP described that “*it really stops you in your tracks to make you reflect on how very important it is to make sure you get the message across*,* because that flash few seconds of anger has resulted in a lifetime’s regret*” (HCP20). Midwives in particular were more likely to deliver messages if they knew an infant with AHT, with some describing colleagues not using ICON if they did not have this experience. Health visitors described ease of adoption due to practicality (e.g. comforting strategies), similarity with pre-existing messages, and congruity with clinical skills. Newer staff incorporated ICON readily, as it was contained in training and embedded in routine tasks. Overall, health visitors adopted ICON more readily and were not as dependent on personal experience as a motivator.

#### Barriers

Managers described organisational challenges such as funding or working across multiple teams with competing priorities. HCPs highlighted issues including inconsistent training strategies (e.g., one-off training, learning on the job, email), high workload, low morale, or staff not seeing relevance. Challenges maintaining adequate staffing affected willingness to adopt, and all HCP groups described challenges completing appointments on time due to the number of other topics to cover.

Only one GP interviewed received training or delivered ICON messages. They described minimal involvement with ICON, only discussing it if parents bring up challenges with crying. All GPs described hesitancy others might feel incorporating ICON into new baby and mother appointments, commenting on insufficient time or concerns that discussing ICON “*might open a can of worms*” (HCP32). GPs described their role as signposting to services rather than addressing crying themselves, often referring to health visitors if there was no medical issue.

*HCP33: If something was going to make the biggest impact*,* it would be how the health visitor delivers these types of interventions. I think it would be a bit over-optimistic and almost arrogant to think me mentioning something for 30 s is going to make a massive difference. How many people actually click on a [webpage] link after I’ve sent it?*

### Implementation of the ICON programme

Implementation questioning explored fidelity to the ICON intervention and its resources.

#### Facilitators

Whilst local adaptations were sometimes required, consistency of implementation across sites was enabled by having a business plan for midwifery and health visiting contact points aligned with ICON touchpoints, involvement of non-health services, availability of promotional materials and technology, and individualising approaches to delivery. Managers commonly perceived messaging was routinely delivered by midwifery and health visiting services but not primary care, consistent with the adoption theme.

*M11: We’ve had a number of rapid reviews*,* so review processes where an infant has been significantly injured*,* and what they are telling us is the ICON message is being delivered in midwifery care and within 0–19 [child health nurse services] but that it’s not being delivered in primary care according to the documentation. It may be being delivered*,* but it’s just not being documented that it is being delivered. So*,* I think the only evidence that we have at the moment in terms of how well embedded it is*,* is via our rapid review processes and they are telling us that.*

Some sites adjusted service plans to deliver ICON at the three Weeks postpartum touchpoint, including through automated text messaging, as many did not previously have this contact. Staff described ICON delivery outside core touchpoints including: 36-week prenatal appointments, NICU for premature babies, visits for feeding concerns, and by DadPad (an organisation supporting new fathers) visiting families in hospital. Managers described the importance of implementing ICON through other services such as childcare centres or police contact with pregnant women and families. One manager commented that they “*didn’t want it just being about health and health services delivering it. We thought it can’t be a success [unless it involves] every professional that could have contact with a family*,* including the police force*” (M8).

Staff were positive about materials developed by the ICON programme, and appreciated the ability to tailor materials to clinical settings, such as leaflets for NICU, waiting room posters, or using the crying curve to normalise crying. Some (though not all) had an insert for the child’s medical record outlining ICON messages. This individualisation of methods was valuable in helping staff consistently deliver ICON messaging, whether electronic (e.g., text messages and websites) or physical materials (e.g., leaflets, posters, or the Red Book). Simplifying the process for staff was crucial.

*M1: I think the thing is we’ll try and adapt and if we can’t do it one way*,* we try and adapt it in a different way. You want to make it as easy as you have said for staff because they won’t take it onboard if it’s too difficult to do. They haven’t got time in their day*,* have they*,* to do things that are too difficult.*

#### Barriers

Staff described challenges implementing ICON, including lack of fidelity to core touchpoints and messages given. Participants described a disconnect between [1] ICON’s recommended touchpoints [2], the scheduled plan of postnatal contact, and [3] when contact with parents actually occurred. For example, the 3-week postpartum ICON touchpoint is not a routine visit for health visitors.

A significant disconnect was identified regarding who delivers messages in practice. ICON recommends detailed discussion by midwives, yet health visitors were frequently frustrated when parents reported not hearing about ICON in hospital or from community midwives. Consequently, health visitors described increased workload due to delivering the entire ICON message rather than a brief recap. Midwives similarly stated some colleagues did not always discuss ICON, citing insufficient time or busy workloads as reasons for supplying the ICON brochure without discussion. However, many managers believed their staff disseminated ICON messages at all recommended touchpoints. This difference between recommendations and practice was evident in many teams.

*HCP28: Quite often the ICON plans that the midwife is supposed to do with them aren’t [done]. I don’t think I’ve ever seen*,* I think I might maybe see a handful. So I don’t think the midwives take it as seriously as we do…. I think the midwives perhaps are not*,* aren’t quite so compassionate about the message.*

Delivery varied between sites, from only giving a video link to actively discussing materials. Some HCPs reported that ICON had at times become a “leaflet campaign” in hospitals, where it is simply handed out or the ICON video was given to the parent(s) to be watched without further discussion. Midwives reported handouts were often not being read due to the high volume of written material for new parents, particularly those with more than one child.

*HCP15: The amount of extra things… we kind of have to do*,* the visits get longer with all the information we have to kind of cover. … You do feel a bit like you’re bombarding sometimes. Have I covered safe sleep*,* have I covered immunisations*,* have I covered dental health*,* have I covered speech and language*,* have I covered the ICON message? You know there’s so many things and you think*,* ‘God*,* I’ve just bamboozled this poor mother.’ Which of these messages will actually stay in her head?*

Limited resources meant some health visitors only carried one copy of the ICON leaflet, showing this during appointments but not leaving physical materials, whilst some described bringing leaflets to appointments as a conversation starter. Others noted inconsistency between staff use of materials and messages delivered, recommending annual training to increase fidelity to the message. One manager (M15) said she was “*interested to see what the consistency is throughout the messages that we’re giving. … I’m not sure everyone’s giving the same message*.”

### Maintenance of the ICON programme

The maintenance dimension explored whether ICON was embedded in policy and clinical practice.

#### Facilitators

Strategic ownership of the programme, managerial support, having a training plan, and having resources readily available facilitated maintenance. Managers described the importance of governance structures in ensuring ICON remained a priority. Successful sustainability strategies included creating and maintaining invested multi-agency steering committees, task and finish groups, attending national meetings to share ideas, and conducting documentation audits. One described the importance of a “*governance structure so that it had that sustainability and ownership from a particular group or department that ensured that it would have the resources behind it to continue it*” (M2).

Ongoing momentum was key, and training helped newer staff get “*confidence up in actually giving the message because it is not an easy message to give out to parents to be fair*” (HCP19 Tracey), and ongoing training maintained impetus and fidelity of message delivery.

*HCP17*: *I think there has to be ongoing training. You can’t just train everyone on the unit once like you can with some things; there has to be refreshers and probably annual updates and to have trainers on the unit that people can go to and one designated person that everybody can go to if they have got a question about it.*

Some health visitors commented on the usefulness of resources on the ICON website such as leaflets in other languages or videos. HCPs stated documenting delivery of ICON fostered maintenance.

*HCP18: I find it’s good to have something that you can tick off because otherwise you don’t wanna talk to parents every day repeatedly about ICON*,* but also you don’t wanna miss it out*,* so something that’s documented to say that you have discussed it is also good so that you know whether it’s been broached before or whether it’s the first time they have been hearing about it. I think everyone should do it*,* definitely.*

#### Barriers

Barriers included limited strategic vision, training or resources, and high workloads. One manager stated the biggest challenges to maintaining momentum are staff-related: “*time*,* resources*,* energy*,* lack of motivation*” (M15). Although managers described the value of governance structures, not all sites had a governance structure in place. One challenge was identifying what portfolio should include ICON and which individual would lead, such as children’s services, safeguarding, or public health.

*M2: We don’t really have that strategic ownership*,* and the system is very reliant on individuals*,* and it isn’t owned by someone within a job description…so when people inevitably are retiring or moving on*,* there’s a risk that the programme won’t be sustained.*

Limited or no regular training on ICON impacted consistency and frequency of ICON delivery. HCPs recommended regular training sessions to combat this.

*HCP1: I think my only concern would be is that because there’s so much health information to share*,* that it doesn’t get diluted maybe*,* but actually*,* it’s a very key*,* important thing to be talking about. I don’t know when the initial training was. Was it three years ago it came out now?*

*M14: Yeah – it’s more like*,* ‘Watch one*,* do one*,* teach one*,*’ unfortunately*,* with these types of information*,* and I think it’s part of the ‘Discharge chat’*,* so if you’ve got a new midwife that hasn’t done ‘Discharge chat*,*’ she’ll watch one midwife do it*,* and then she’s off doing her… ‘Discharge chat’ next. So*,* there isn’t any annual education about this topic.*

Participants described challenges related to a regional rather than national approach to ICON adoption. HCPs commented that neighbouring communities varied in ICON implementation, including one midwife describing that a serious head injury occurred in a neighbouring community where ICON was not mandated. Staff described the value a national campaign would have on increasing awareness of ICON, thus normalising it within broader society. This would benefit new parents and improve staff buy-in and engagement. Participants gave the example of ICON Week [21] as a successful national campaign, occurring annually in September. HCPs described how ICON Week provided the opportunity to increase awareness of the ICON message around the unit or clinic.

## Discussion

**Fig. 1:**
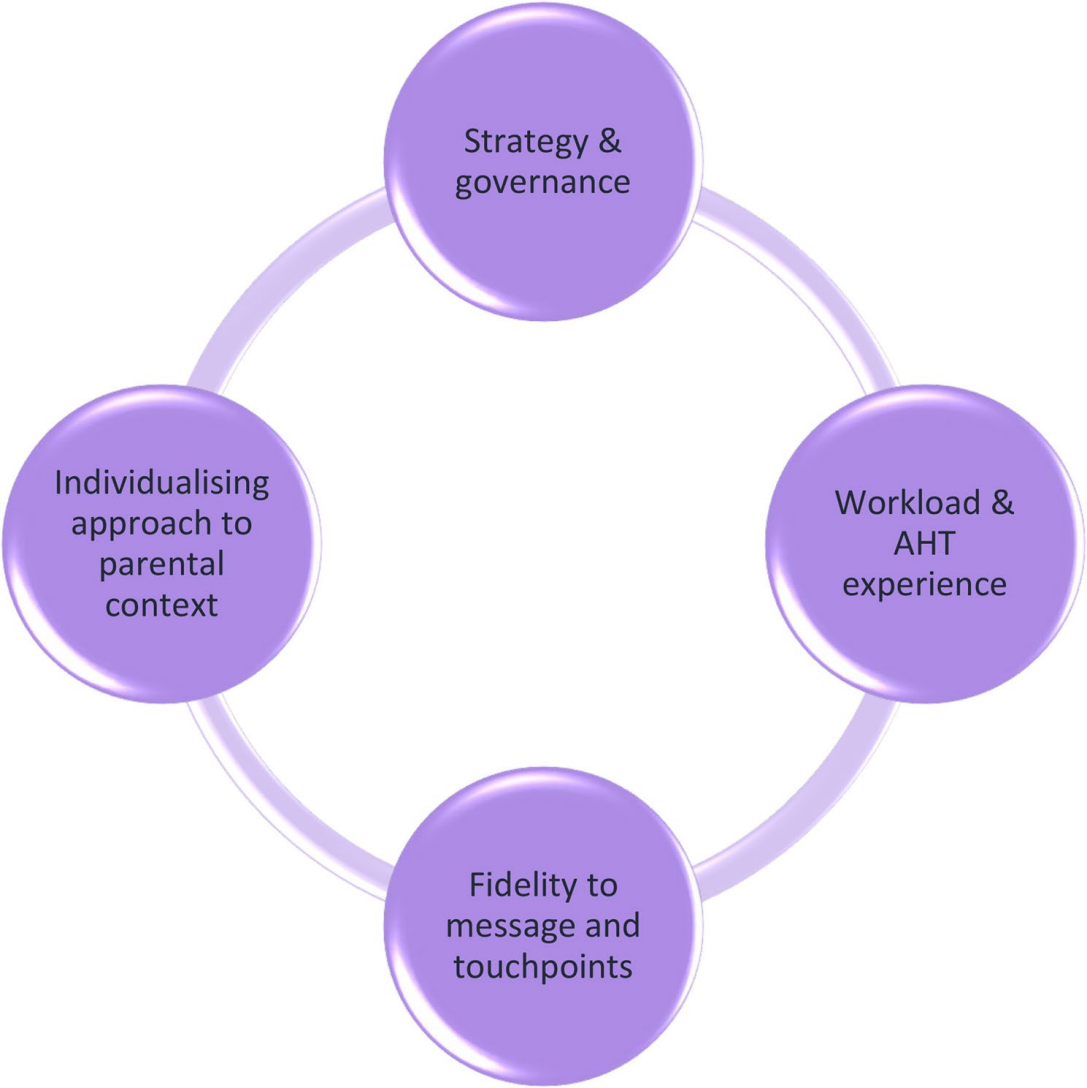
Enablers and challenges to the effectiveness of the ICON programme

This study evaluated the ICON programme from the perspective of managers and HCPs through the RE-AIM framework using qualitative methods. It is the first study to explore key enablers and obstacles to the reach, effectiveness, adoption, implementation, and maintenance of the ICON programme. As an intervention’s impact needs to be explored within the context of “real life”, staff involved in commissioning and delivering the ICON message were interviewed to highlight any barriers or facilitators [19, 22]. The four main enablers and related challenges regarding the ICON programme identified by participants across multiple RE-AIM domains were (Fig. 1): (a) reach affected by fidelity to the programme’s recommended touchpoints and message; (b) individualised approaches to address contextual factors impacted parental receptiveness; (c) staff buy-in was related to workload, previous AHT experience, and training; and (d) ensuring a strategic role and governance structure fostered successful adoption, implementation, and maintenance.

First, participants described significant variation in the number of touchpoints delivered, information discussed, and format of that delivery. This likely affects the ability to reach the intended audience and effectiveness in preventing AHT and may impact future evaluations of ICON. Factors affecting fidelity of implementation therefore need to be addressed. Adherence to touchpoints and how a message is delivered are not routinely explored in research on AHT prevention education. However, the significance of good interprofessional communication when providing patient education is highlighted in other research, as it affects reach and effectiveness of an AHT prevention programme [23]. One study reported that education provided by HCPs was no more effective than other forms of dissemination, such as in news/media or from a friend, yet reasons for this were not explored [9].

Second, staff who individualised approaches with new parents when delivering ICON messages described greater parental interest in ICON. Ensuring fidelity to ICON messages, HCPs used clinical judgement and prior experience to know how to do so, leading to the perception they were reaching more parents more effectively. Similarly, effective interprofessional communication on specific parental needs when transferring parents between services is essential between midwives, health visitors, and GPs. Many studies on AHT prevention do not discuss how delivery methods impact effectiveness; however research on clinical education for patients highlights the importance of ensuring the message is relevant to the individual and ensuring good interdisciplinary communication [24, 25]. For example, participants who described strategies to reach fathers or male carers were more effective in reaching this population group. Unfortunately, participants described relying heavily on mothers passing the ICON information on to their partners, despite an awareness that male carers are the primary perpetrators of AHT.

Third, HCPs described how challenges of high workloads and time constraints delivering multiple health promotion messages impacted ability to deliver ICON messages. Managers and HCPs adopted ICON more readily when they had experienced a case of AHT or were involved in serious case reviews, as this emphasised its importance and motivated staff to deliver ICON messages. Time constraints are an ongoing professional concern; a Royal College of Midwifery survey of Heads of Midwifery reported workforce shortages of 87% in 2021, compared to 71% in 2020 [26]. Consistent with other studies, a primary reason for limited educational message delivery is the amount of time required to deliver the material within the short time allocated [27]. Education and training help HCPs remember key ICON messages, and regular refresher training time needs to be protected for staff with high workloads.

Fourth, strategic ownership and collaborative governance fostered adoption, implementation, and maintenance of the ICON programme. Commissioners, designated safeguarding leads, and managers were the primary strategic leads addressing AHT in infants across the healthcare system. Strategic roles included preventative actions such as: adopting and implementing the ICON programme; working with healthcare providers and stakeholders; obtaining funding for set-up and maintenance; developing business plans for sustainability; ensuring ownership at all levels; delivering full and regular training for staff; and incorporating ICON reporting into documentation standards. Strategic roles also included reactive measures such as reviewing cases of infant injury or death, identifying lessons learned, and gaps in services. Steering groups and interagency partnerships helped maintain momentum for ICON over time, working towards shared goals. Although the significance of strategic ownership and collaborative governance on the effectiveness of educational programmes on AHT in infants is infrequently discussed in existing literature, a growing body of research highlights the importance of collaborative governance in other public health campaigns [28].

### Strengths and limitations

A primary strength of this study was the large number of participants including various professions and grades, enabling capture of a broad range of views. Recognising the importance of input from multiple staff groups during the initial recruitment phase, we exceeded the anticipated sample size to include commissioners, managers, safeguarding leads, midwives, nurses, health visitors, GPs, and infant feeding teams. This approach garnered a comprehensive picture of the context of ICON roll-out in the regions studied. Another study strength was obtaining perspectives on an educational intervention across multiple regions in England, including from the north, central and south of England and communities with varied cultural backgrounds and supporting clients experiencing different levels of advantage. Online interviews and focus groups facilitated data collection across England, booked at times convenient to healthcare teams. Following data analysis, we ran a stakeholder event to discuss findings with managers and HCPs involved in the study; themes were positively validated, and no participants expressed contradictory views. There was unanimity in affirming the importance of a national strategy addressing AHT. One potential limitation relates to the challenge of recruiting midwives and GPs, which may be due to lower use of ICON in practice and limited capacity to participate; only three GPs and four midwives were interviewed. In addition, there was limited cultural and gender diversity of participants in the study, reflecting limited diversity in the workforce (e.g., less than 0.3% of midwives are male) [29]. Limited ethnic and gender diversity of the workforce is a concern, as recognising issues specific to families from diverse ethnic groups and men may not have been noted.

## Conclusion

In this study evaluating the ICON AHT prevention programme, key barriers and facilitators to delivery, impact and sustainability were identified, with validity of findings enhanced by taking a system-wide approach including commissioners, managers, health visitors, midwives, nurses, GPs, and other staff delivering ICON messages. Prioritisation of the ICON programme by leadership staff impacted adoption, implementation and maintenance. Healthcare professionals are willing and able to deliver messages to new parents, but require training and time to do so.

Understanding barriers and facilitators to delivery can inform policy by guiding processes to facilitate adoption, implementation, and maintenance of the ICON programme. This awareness is crucial when identifying whether a programme is reaching the intended client group, and ultimately achieving the stated aim of reducing the incidence of infant AHT.

## Supplementary Information

Supplementary Material 1.

## Data Availability

Requests to access anonymised transcript data should be made to the corresponding author and all appropriate requests will be considered.
