# Supplementary material for: Healthcare leaders and professionals’ perspectives of the ICON programme to prevent abusive head trauma in infants: a qualitative study
Source: BMC Public Health. 2025 Oct 21;25:3541. doi: 10.1186/s12889-025-24682-0 (PMC12538752; doi:10.1186/s12889-025-24682-0)
Supplement: Supplementary file 1 — Supplementary Material 1. [file 12889_2025_24682_MOESM1_ESM.docx]

**Additional File 1**

| Table 1. Sample questions from the topic guide: Leadership interviews |  |
| --- | --- |
| **Sample interview questions** | **RE-AIM concept** |
| When you first heard of the ICON programme, what were your initial thoughts? | Adoption |
| Could you describe your experience with the process of setting up the ICON programme?  What were the challenges implementing the ICON programme at your site? Other sites?  On reflection, was there anything you would have done differently when rolling out the programme? | Implementation |
| How well established is the ICON programme in your service?  Will your site continue to use the ICON messages in the future?  What do you think are the benefits for continuing the ICON messaging?  What challenges can you foresee in continuing to deliver the ICON programme?  What resources and support do you feel are needed to continue to embed the intervention? | Maintenance  Effectiveness |
| Overall, what are your thoughts on the programme?  What recommendations do you have for other sites who want to implement the ICON program? |  |

| Table 2. Sample questions from the topic guide: HCP interviews |  |
| --- | --- |
| **Sample interview questions** | **RE-AIM concept** |
| When you first heard of this project, what were your initial thoughts about ICON?  Was there organisational support to add the ICON programme to your workload?  Was there any pushback from your colleagues or yourself about not wanting to participate? | Adoption |
| Could you describe your experience with the process of implementing ICON?  What ICON materials do you use, and how do you use them with your parents?  How has your message to parents about crying babies changed since ICON was rolled out?  What do you think is helpful about the ICON intervention, advice and resources?  What have been the challenges or what has worked well in delivering the ICON programme to parents and carers?  Are there any changes you would find helpful in the material? | Implementation  Reach  Effectiveness |
| Since ICON began, do you feel you are having more conversations about how to deal with a crying baby with your patients, using ICON material?  What further resources and support would be helpful to sustain and further embed the ICON messages in your service? | Reach  Maintenance |
